# MR Imaging Evaluation of Intracerebral Hemorrhages and T2 Hyperintense White Matter Lesions Appearing after Radiation Therapy in Adult Patients with Primary Brain Tumors

**DOI:** 10.1371/journal.pone.0136795

**Published:** 2015-08-31

**Authors:** Dong Hyun Yoo, Sang Woo Song, Tae Jin Yun, Tae Min Kim, Se-Hoon Lee, Ji-Hoon Kim, Chul-Ho Sohn, Sung-Hye Park, Chul-Kee Park, Il Han Kim, Seung Hong Choi

**Affiliations:** 1 Department of Radiology, Seoul National University Hospital, Seoul, Korea; 2 Department of Neurosurgery, Seoul National University Hospital, Seoul, Korea; 3 Department of Internal Medicine, Cancer Research Institute, Seoul National University Hospital, Seoul, Korea; 4 Department of Pathology, Seoul National University Hospital, Seoul, Korea; 5 Department of Radiation Oncology, Cancer Research Institute, Seoul National University Hospital, Seoul, Korea; Wayne State University, UNITED STATES

## Abstract

The purpose of our study was to determine the frequency and severity of intracerebral hemorrhages and T2 hyperintense white matter lesions (WMLs) following radiation therapy for brain tumors in adult patients. Of 648 adult brain tumor patients who received radiation therapy at our institute, magnetic resonance (MR) image data consisting of a gradient echo (GRE) and FLAIR T2-weighted image were available three and five years after radiation therapy in 81 patients. Intracerebral hemorrhage was defined as a hypointense dot lesion appearing on GRE images after radiation therapy. The number and size of the lesions were evaluated. The T2 hyperintense WMLs observed on the FLAIR sequences were graded according to the extent of the lesion. Intracerebral hemorrhage was detected in 21 (25.9%) and 35 (43.2) patients in the three- and five-year follow-up images, respectively. The number of intracerebral hemorrhages per patient tended to increase as the follow-up period increased, whereas the size of the intracerebral hemorrhages exhibited little variation over the course of follow-up. T2 hyperintense WMLs were observed in 27 (33.3%) and 32 (39.5) patients in the three and five year follow-up images, respectively. The age at the time of radiation therapy was significantly higher (p < 0.001) in the patients with T2 hyperintense WMLs than in those without lesions. Intracerebral hemorrhages are not uncommon in adult brain tumor patients undergoing radiation therapy. The incidence and number of intracerebral hemorrhages increased over the course of follow-up. T2 hyperintense WMLs were observed in more than one-third of the study population.

## Introduction

Various complications, ranging from asymptomatic to fatal, occur in the central nervous system after radiation therapy of the brain, and magnetic resonance (MR) imaging is useful in detecting these complications [1,2]. Intracerebral hemorrhages and T2 hyperintense white matter lesions (WMLs) are frequently encountered complications on follow-up MR imaging. Hemorrhagic complications likely result from radiation-induced telangiectasia, a type of microangiopathy otherwise referred to as cryptic vascular malformation, which is caused by radiation-induced endothelial injury and subsequent venous occlusion [3,4]. Hyperintense WMLs on T2-weighted or fluid-attenuated inversion recovery (FLAIR) T2-weighted images are caused by radiation-induced demyelination [5]. Although these complications have been reported previously, the majority of these studies were conducted on pediatric patients with limited study populations and variable follow-up periods [6–8].

The purpose of this study is to evaluate the brains in a relatively large number of adult primary brain tumor patients previously treated with radiation using FLAIR T2-weighted and gradient echo (GRE) sequence MR images. We aimed to determine the incidence and severity of intracerebral hemorrhages and T2 hyperintense WMLs over a specific period of time following radiation therapy for brain tumors in adult patients.

## Materials and Methods

### Ethics Statement

This retrospective study was approved by the institutional review board of Seoul National University College of Medicine and Hospital, and the need for written informed consent was waived. The patient name and study date was not de-identified prior to the image analysis. However, other patient information was blind to the radiologists.

### Study Population

Between 1983 and 2009, 648 adult patients (age of 18 years or older) with newly diagnosed primary brain tumors received radiation therapy of the brain. From the original study population, we selected 88 patients for whom MR images consisting of a GRE and FLAIR T2-weighted sequence were available for three years and five years following the radiation therapy. An additional seven patients were excluded because extensive tumor progression prevented analysis of the MR images. As a result, 81 patients (44 males and 37 females) with a mean age of 39.7 years (range 22 to 69 years, SD ±10.0) at the time of the radiation therapy were enrolled as the final study population for analysis. Among the study population, 48 patients underwent a brain MR study seven years after radiation therapy, and these MR images were also reviewed as well. The pathological diagnoses included astrocytoma (n = 3), anaplastic astrocytoma (n = 19), anaplastic oligoastrocytoma (n = 5), oligodendroglioma (n = 16), anaplastic oligodendroglioma (n = 34), ependymoma (n = 2), subependymoma (n = 1) and anaplastic ganglioglioma (n = 1).

### Brain Radiation Therapy and Clinical Data

Radiation therapy was initiated 2 to 4 weeks following surgery. Conventional external field radiation in fractions of 1.8–2.0 Gy 5 times a week was performed with a mean total dose of 58 Gy (range: 46.8–61.2 Gy), using 4-6MV x-rays generated from a linear accelarator. The 3-dimensional radiotherapy technique with a multileaf collimator (MLC) system was utilized after optimization by a radiotherapy planning system. The field size was determined to encompass a 1.5 cm margin from the tumor bed or gross tumor initially and a 5 mm margin after field reduction, with an additional 3 mm margin for possible physical error. The dose constraint was 54 Gy for the brainstem, 54-58Gy for the optic apparatus, and 45–50 Gy for the upper cervical cord. All the patients minus one underwent involved-field radiation therapy, and one patient with an infratentorial ependymoma received whole brain irradiation. Chemotherapy was administered in 63 patients as follows: before radiation therapy in 16 patients, after radiation therapy in 41 patients, and concurrent with radiation therapy in 6 patients. The medical records were reviewed for other data, such as the clinical course and adverse radiation effects. The end of the follow-up period was defined as December 2012. For the patients who were lost to follow-up, the end of the follow-up period was defined as the most recent date on which follow-up information was obtained.

From the medical records, we analyzed the symptoms known to be common adverse radiation effects, including the following: headache, dizziness, somnolence, skin problems, cognitive dysfunction, and newly developed seizures. In patients with tumor progression or hydrocephalus, we analyzed the data before these events or the onset of symptoms related to these events for symptomatic analysis.

### MR Imaging and Evaluation

The MR images were obtained using 1.0 T (Magnetom Expert, Siemens), 1.5 T (Signa, GE Healthcare, Milwaukee, WI; or Magnetom Vision Plus, Siemens) or 3T (Signa, GE Healthcare; or Magnetom Verio, Siemens) MR imaging system. The MR imaging sequences included FLAIR T2-weighted images (TR/TE/NEX, 8802–9902/119–164/1, 5-mm-thick sections, 1.0–2.0-mm intersection gap) and GRE sequence images (TR/TE/NEX, 467-800/20-26/1, 5-mm-thick sections, 1.0–2.0-mm intersection gap).

The images were evaluated by two independent radiologists (S.H.C. and D.H.Y., with 12 and 6 years of experience in neuroradiology, respectively), and the final decision on discrepant results was reached by mutual consent. The MR imaging data within a one-year range closest to the three- and five-year follow-up date after the start of the radiation therapy were used for the analysis. Pre-treatment FLAIR T2-weighted images were available for all the patients, whereas pre-treatment GRE images were available for 47 patients.

The MR imaging data of the brain following radiation therapy were serially reviewed to detect intracerebral hemorrhages. Intracerebral hemorrhage was defined as a hypointense dot lesion on GRE sequence MR images. Lesions in the basal ganglia and thalamus, which is known to be a predominant location for intracerebral hemorrhages in general population, were excluded. Additionally, those adjacent to the surgical margins were excluded. The number and size of the lesions were evaluated. The lesions were subdivided into the following three groups according to size of the patient’s largest lesion; less than 5 mm (small), 5–9 mm (medium), and larger than 9 mm (large).

T2 hyperintense WMLs observed on the FLAIR sequences were graded according to a modification of the system of Wilson et al [7,9]. Grade 1 was defined as patchy, mildly increased signal intensity in the periventricular white matter; grade 2 as moderate changes that extended almost to the gray-white junction, sparing the subcortical U-fibers; and grade 3 as severe changes, confluent from the level of the frontal horns to that of the trigones, with or without involvement of the U-fibers. The MR images prior to radiation therapy were carefully reviewed to exclude preexisting hyperintensity related to peri-tumoral edema, infiltration of tumor, and postoperative changes.

### Statistical Analysis

The SPSS 17.0 package (SPSS Inc., Chicago, IL) was used for the statistical analysis. The Student’s *t*-test was used for the comparison of the continuous variables (age) between the patients with and without lesions. The number and size of the intracranial hemorrhages between the three- and five-year follow-up data were also compared using the Student’s *t*-test. The categorical values (gender, chemotherapy, symptoms) were compared using a chi-square test. The cumulative incidence rates were determined by Kaplan-Meier estimation. The results with a *p*-value of less than .05 were considered statistically significant. No correction for p-value was performed.

## Results

### Intracerebral hemorrhage

**Fig 1:**
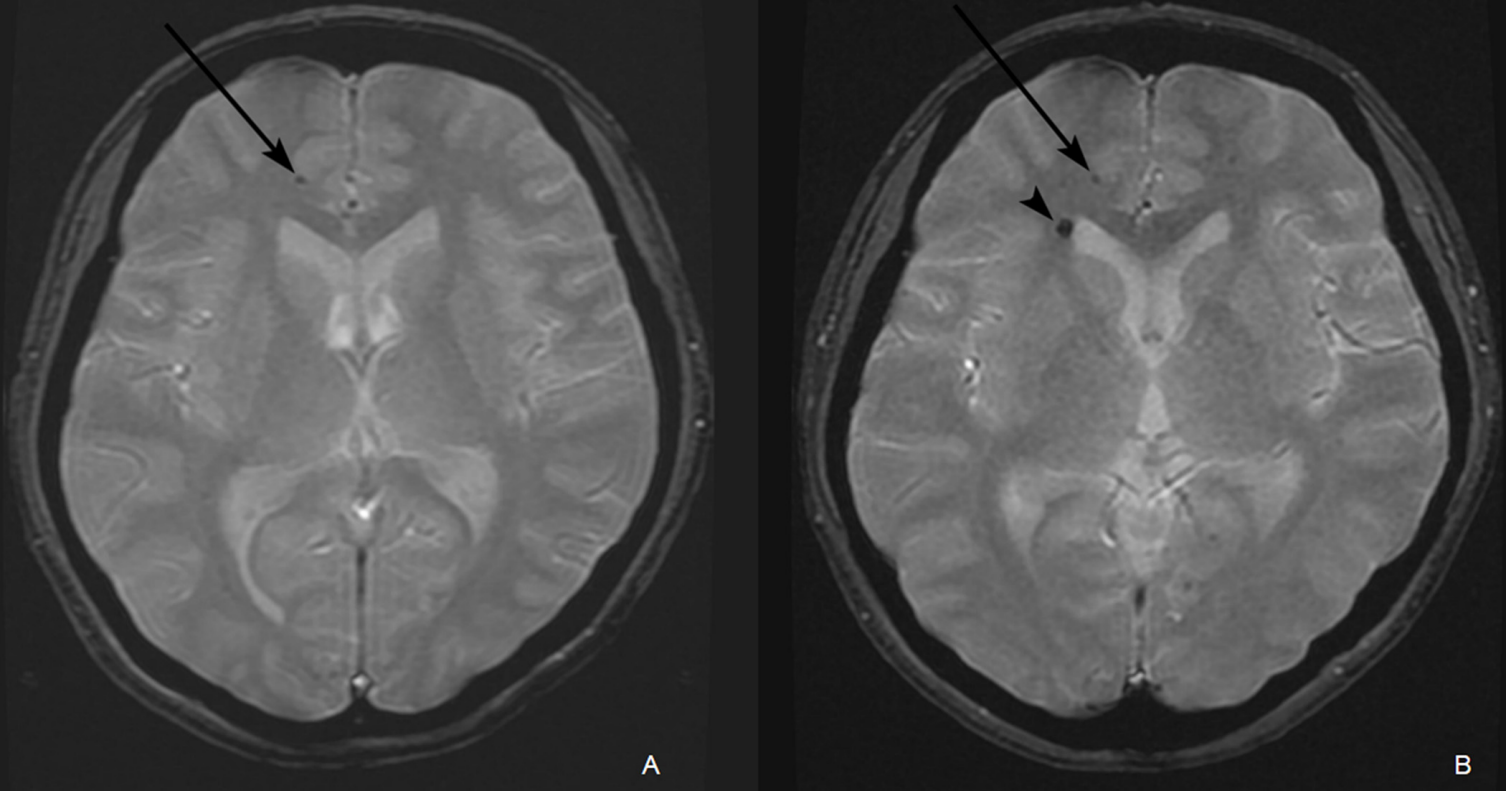
A of a 34-year-old female who received radiation therapy due to an ependymoma in the fourth ventricle. (A) A gradient echo (GRE) image acquired five years following radiation therapy revealed a small dark dot lesion (arrow) in the subcortical white matter of the right frontal lobe. (B) A GRE image acquired seven years following radiation therapy revealed a newly developed intracerebral hemorrhage (arrowhead) adjacent to the right lateral ventricle frontal horn next to the previously noted intracerebral hemorrhage in the subcortical white matter of right frontal lobe (arrow).

**Fig 2:**
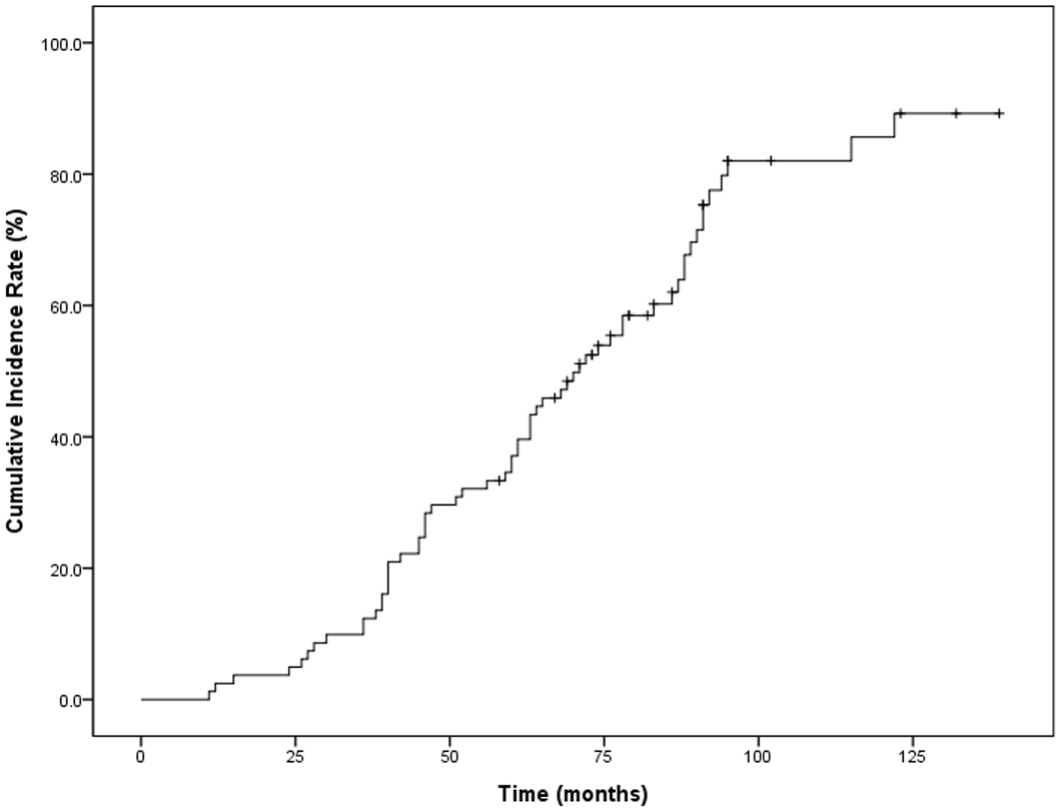
Kaplan-Meier estimation of the cumulative incidence of intracerebral hemorrhage.

A typical case of intracerebral hemorrhage on GRE image is presented in Fig 1. The mean follow-up period was 96.5 months (SD, 24 months; range, 58–152 months), and intracerebral hemorrhage appeared in 60 of 81 patients (74.1%). The shortest follow-up interval in which an intracerebral hemorrhage was detected was 11 months, whereas the longest interval was 122 months (mean, 59.7 ± 25.1 months). The cumulative incidence rates of intracerebral hemorrhage after radiation therapy were 2.5% at one year, 12.3% at three years, 37.1% at five year, 62.0% at seven years, and 89.2% at ten years (Fig 2).

Table 1 summarizes the incidence, numbers, and sizes of the intracerebral hemorrhages following radiation therapy. Between the three- and five-year follow-up interval, 17 patients developed new intracerebral hemorrhages. In three patients, intracerebral hemorrhages were detected on the three-year MR images; however, there was no intracerebral hemorrhage on the five-year MR images. Among the 35 patients with intracerebral hemorrhages on the seven-year MR images, 15 patients had no intracerebral hemorrhages on the previous MR images. In three patients, intracerebral hemorrhages were detected on the five-year MR images, with no intracerebral hemorrhage found on the seven-year MR images. Among the 35 patients with an intracerebral hemorrhage on the five-year MR images, 12 patients did not have seven-year MR images.

**Table 1:** Presence, size, and number of intracerebral hemorrhages of the brain.

	3 year (n = 81)	5 year (n = 81)	7 year* (n = 48)
Patient with intracerebral hemorrhages, n (%)	21 (25.9)	35 (43.2)	35 (72.9)
Size^†^, n (%)			
Small	18 (22.2)	30 (37.0)	28 (58.3)
Medium	2 (2.5)	5 (6.2)	5 (10.4)
Large	1 (1.2)	1 (1.2)	2 (4.2)
Number^‡^, n (%)			
1	13 (16.0)	13 (16.0)	3 (6.3)
2 to 9	7 (8.6)	18 (22.2)	27 (37.5)
10 or more	1 (1.2)	4 (4.9)	5 (10.4)

* Based on 48 patients for whom MR image data were available 7 years after brain radiation.

^†^ If a patient had more than one hemorrhage, the one with the largest diameter was measured.

^‡^ Numbers of hemorrhages in one patients

The number of intracerebral hemorrhages per patient tended to increase as the follow-up period increased, whereas the size of the intracerebral hemorrhages exhibited little variation in the course of follow-up. Among the patients with intracerebral hemorrhages, the mean and median number of intracerebral hemorrhages was 2.0 and 1 on the three-year MR images and 6.1 and 3 on the five-year MR images (*p* = 0.048 for the mean values). The largest intracerebral hemorrhage measured was 11 mm on the three- and five-year MR images and 12 mm on the seven-year MR images. The development of intracerebral hemorrhages showed no preponderance of location.

The incidence of intracerebral hemorrhages did not significantly differ between genders on the three-year (male vs. female; 27.3% vs. 24.3%, *p* = 0.763) or five-year (45.5% vs. 40.5%, *p* = 0.657) follow-up MR images. Furthermore, there was no significant difference in age at the time of radiation therapy between the patients with intracerebral hemorrhages and those without: 36.9 vs. 40.7 (*p* = 0.129) on three-year and 38.3 vs. 40.8 (*p* = 0.275) on five-year follow-up. The difference in the incidence of intracerebral hemorrhages between the patients who received chemotherapy and those who did not was not significantly different on the three-year (28.6% vs. 16.7%, *p* = 0.309) and five-year (47.6% vs. 27.8%, *p* = 0.134) images.

### T2 hyperintense WMLs

The number of patients exhibiting a T2 hyperintense WML is presented in Table 2 according to the grading. In eight patients, there was a progression of grade as follows: four patients from grade 0 to 1, three patients from grade 1 to 2, and one patient from grade 0 to 2. No patient exhibited downgrading of T2 hyperintense WMLs. In the three- and five-year follow-up MR images, the age at the time of radiation therapy was significantly higher (*p* < 0.001) in the patients with T2 hyperintense WMLs than in those without WMLs (36.5 vs. 46.1 on the three-year and 36.2 vs. 45.0 on the five-year follow-up). There was no significant correlation between the T2 hyperintense WMLs and gender or the administration of a chemotherapeutic agent.

**Table 2:** Number of patients with a T2 hyperintense white matter lesion on a FLAIR T2-weighted image.

	3 year	5 year
Grade, n (%)		
1	13 (9.8)	14 (10.5)
2	12 (9.0)	16 (12.0)
3	2 (1.5)	2 (1.5)
Total	27 (33.3)	32 (39.5)

### Symptomatic analysis after radiation therapy

The median clinical follow-up period was 91 months (range, 58–152). The median period to disease progression or malignant transformation was 66 months (range, 18–117) in 26 patients, and ventriculo-peritoneal shunts were performed 27, 54, 78, 84, and 129 months after radiation therapy in 5 patients. After excluding the clinical adverse effects that occurred after the events mentioned above for the symptomatic analysis, the median follow-up period was 83 months (range, 18–152).

The possible clinical adverse effects after radiation therapy and the time interval from radiation therapy to symptom onset are presented in Table 3. The incidence of each symptom did not significantly differ between the patients with and without intracerebral hemorrhages. The time interval from radiation therapy to the onset of various symptoms was shorter than the time interval to intracerebral hemorrhage, which represented a 70-month median interval from radiation therapy.

**Table 3:** The incidence of adverse symptomatic radiation effect.

	Total 81, n (%)	Microhemorrhage +, n (%)	Microhemorrhge–, n (%)	*p*-value^a^	Time interval^b^
Headache	43 (53.1)	30 (50)	13 (61.9)	0.448	15 (3–129)
Dizziness or vertigo	20 (24.7)	12 (20)	8 (38.1)	0.140	16.5 (3–78)
Sleepiness	12 (14.8)	10 (16.7)	2 (9.5)	0.722	30 (3–78)
Skin problem	13 (16.0)	8 (13.3)	5 (23.8)	0.305	15 (3–51)
Cognitive dysfunction	24 (26.9)	20 (33.3)	4 (19.0)	0.274	25.5 (6–129)
Newly developed Seizure	9/63^c^ (14.3)	8/47^c^ (21.3)	1/16^c^ (6.3)	0.434	48 (9–108)
Language dysfunction	5 (6.2)	5 (8.3)	0 (0)	0.320	15 (3–144)
Motor weakness	13 (16.0)	9 (15.0)	4 (19.0)	0.733	30 (9–90)
Sensory change	3 (3.7)	2 (3.3)	1 (4.8)	1.000	36 (30–51)
Blurred vision	8 (9.9)	8 (13.3)	0 (0)	0.104	24 (6–114)

^a^ Statistical significance of the difference in the incidence of each symptom between the patients with an intracerebral hemorrhage and without an intracerebral hemorrhage

^b^ Time interval from radiation therapy to symptom onset, median months (range)

^c^ Eighteen patients who experienced seizure before radiation therapy were excluded from this analysis.

## Discussion

Radiation therapy plays a major role in the treatment of brain tumor patients. Various randomized trials have demonstrated a significant survival benefit from post-operative radiation therapy in patients with a newly diagnosed malignant glioma [10]. Additionally, early post-operative radiation therapy increases the progression-free survival in low-grade glioma patients [11,12]. However, radiation injury of the brain after cranial irradiation is a dose-limiting factor that ranges from subclinical changes only detected on MR images to fatal complications such as brain necrosis [1]. Vascular damage is a major contributor to changes or complications in the brain after radiation therapy. Radiation-induced occlusive large vessel vasculopathy occurs in large arteries, such as the internal carotid artery or middle cerebral artery, and might result in Moyamoya vascularity. The latent period has been reported to be as long as 20 years [13]. No patient developed large vessel vasculopathy in our study.

An intracerebral hemorrhage is a result of changes in the microvascular structure after radiation; thrombosis in small venules induces dilatation of the capillaries in an attempt to develop collateral vessels, and a telangiectasia forms [3]. These telangiectasias are prone to subclinical hemorrhage, and subsequent perivascular calcification and hemosiderin deposition are detected as hypointense dot-like lesions on GRE images. A previous study on survivors of pediatric acute lymphoblastic leukemia (ALL) patients who were treated with brain radiation therapy reported a 55% prevalence of intracerebral hemorrhages on GRE images [7]. The mean interval from diagnosis was 12.2 years (range: 5.0–18.8) in the study. In our study, the prevalence was 25.9% over three years, 43.2% over five years, and 72.9% over seven years, whereas the cumulative incidence rates were 2.5% at one year, 12.3% at three years, 37.1% at five year, 62.0% at seven years, and 89.2% at ten years. We suspect that the somewhat higher incidence in our study could be attributed to the natural incidence of intracerebral hemorrhages. In a large study conducted on all the brain MRIs at single center over three years, the incidence of intracerebral hemorrhage was 9.8% on the GRE images. In this study, the detection of intracerebral hemorrhages increased with age, and intracerebral hemorrhages were usually observed in patients older than 40 years [14]. Among the patients with intracerebral hemorrhage in our study, a few might have developed intracerebral hemorrhages independent of radiation therapy.

Most of the intracerebral hemorrhages detected in our study were smaller than 10 mm in diameter. Of the patients with intracerebral hemorrhages, 80% or more had small (less than 5 mm) intracerebral hemorrhages on three-, five- and seven-year follow-up MR images. The number of intracerebral hemorrhage per patient was less than ten in the majority of the patients. The number of intracerebral hemorrhages tended to increase with longer follow-up, possibly as the result of accumulation and new development of intracerebral hemorrhages over time, as has been described in a previous study [7]. The greatest number of intracerebral hemorrhages detected in a patient was 10 and 67 on the three- and five-year follow-up, respectively.

An autopsy study has suggested that immature brains might be more sensitive to radiation [15]. One previous study in pediatric patients who received brain radiation reported a higher incidence of telangiectasias on the T2-weighted MR images of younger patients [6]. Because our study subjects were adults with mature brains, there was no significant age difference between those with and without intracerebral hemorrhages. Additionally, there was no significant difference in the incidence of intracerebral hemorrhages between the group that did and the group that did not undergo chemotherapy in our study. This finding is in accordance with results from previous studies on pediatric patients [6,7].

The clinical implication of radiation-induced intracerebral hemorrhages remains uncertain. In our study, the symptoms after radiation therapy appeared to be unrelated to intracerebral hemorrhages. Intracerebral hemorrhages on the GRE images appeared much later than the symptoms after radiation therapy according to the chronological analysis, and there was no significant difference in the incidence of each symptom between the patients with intracerebral hemorrhages and those without intracerebral hemorrhages.

In a study performed on stroke patients of various subtypes, the presence of intracerebral hemorrhages on GRE images was an indicator of increased risk for bleeding [16]. Therefore, among patients who received radiation therapy, those with intracerebral hemorrhage might be more susceptible to large intracranial hemorrhages than those without intracerebral hemorrhage. In our study, intracerebral hemorrhages were detected in both patients who presented with large intracranial hemorrhages. One patient had 41 intracerebral hemorrhages on the GRE images acquired two years before the hemorrhagic event, whereas another patient had 8 intracerebral hemorrhages one year before the event. In these two patients, the onset of large intracranial hemorrhage was 8 years and 9 years after the radiation therapy. This result is comparable to the mean of 8.1 years (range 1 to 19 years) observed in a previous study in which the authors described 20 cases of large intracranial hemorrhages in patients treated with radiation therapy for central nervous system neoplasia in childhood [17].

Unlike intracerebral hemorrhages, the clinical implication of radiation-induced WMLs is well documented. The extent of T2 hyperintense WMLs after radiation of the brain has been reported to be associated with cognitive dysfunction [18–20]. Radiation-induced WMLs have been widely investigated in various studies and frequencies of as low as 5% to as high as 100% have been reported [1,5,7,19]. To our knowledge, no previous study has described the incidence of T2 hyperintense WMLs as a function of time after radiation therapy. In our study, the incidence of T2 hyperintense WMLs was 33.3% and 39.5% in three- and five-year follow-ups, respectively. Although our study revealed stasis or progression of T2 hyperintense WMLs over the course of follow-up, a decrease or resolution of WMLs has been observed. A study of temporal lobe injury after radiation therapy for nasopharyngeal cancer revealed a decrease or resolution of T2 hyperintense WMLs in approximately 28% of the lesions [21].

The age at the time of radiation therapy was higher in the patients with T2 hyperintense WMLs in our study. As the natural prevalence and severity of WMLs increases with advancing age, this finding might be a result of the aging process, and differentiation of WMLs from old age versus those from radiation was not possible in our study [22,23]. A previous study demonstrated more marked white matter changes with advancing age among those who received radiation therapy compared with the control group [24]. We hypothesize that the T2 hyperintense WMLs in our study are much more attributable to radiation therapy. It is possible that radiation therapy in the brain might cause small and subclinical infarcts by injuring and obstructing the small vessels, thus accelerating normal white matter change.

There are several limitations in our study. First, there are various limitations in our MR imaging data used for detecting intracerebral hemorrhages. Because a considerable number of the intracerebral hemorrhages were small, a few intracerebral hemorrhages might have been present in the intersection gap and not scanned in our MR imaging data. Thus, in three patients, intracerebral hemorrhages were detected on the three-year MR images and not on the five-year MR images. We might have underestimated the true incidence of intracerebral hemorrhage. Additionally, we used MR imaging systems of various magnetic field strengths throughout the study. Recently, susceptibility-weighted imaging (SWI) has been widely available, with improved sensitivity in identifying vascular structures and malformations compared with that of conventional imaging [25–27]. Using SWI might reveal increased detection of intracerebral hemorrhages, which requires further investigation. Second, our study is subject to selection bias because we excluded the patients for whom no appropriate MR imaging data were available. The incidence of intracerebral hemorrhages and T2 hyperintense WMLs in patients with short survival (of less than 5 year) or extensive disease could not be determined. Our Kaplan-Meier estimation of the cumulative incidence of intracerebral hemorrhages suffers from incomplete data because a number of patients recruited earlier did not undergo GRE imaging within three years following radiation therapy. Pre-radiation therapy GRE images were not available in 33 patients, and, although the possibility is low, it is possible that the intracerebral hemorrhages we detected in these patients were present before the radiation therapy. Third, the retrospective nature of this study and the lack of a questionnaire survey or quantitative test inevitably limits thorough symptomatic analysis and could result in underestimation of the outcomes. Close evaluation of cognitive function in patients receiving radiation therapy could reveal clinical effect of intracerebral hemorrhage in the future.

Despite these limitations, this study provides valuable information on two complications following radiation therapy in adult brain tumor patients. Intracerebral hemorrhage is not uncommon in adult brain tumor patients following radiation therapy and the incidence and number of intracerebral hemorrhages increased over the course of follow-up. T2 hyperintense WMLs are not uncommon, and their incidence increases with advancing age.
